# Dental Therapeutics

**Published:** 1865-01

**Authors:** H. S. Chase


THE
### DENTAL REGISTER OF THE WEST.

VOL. XIX.] JANUARY, 1865 [No. 1.

### Original Essays and Communications.

### DENTAL THERAPEUTICS.

Read before the Iowa State Dental Society, at Des Moines, January 4th, 1865.

BY H. S. CHASE.

The importance of a thorough knowledge of the Healing Art, as applicable to the Profession of Dentistry, can hardly be over-estimated. True, the range of subjects to which it *seems* to be limited, is *not* extensive, compared with the whole field of Medical Science, yet the two are so intimately connected that the Dental Student can hardly get a thorough knowledge of his own proper department without drawing largely from the same fountain that has supplied his brother in the medical profession.

Without good attainments in Dental Therapeutics, the practitioner must sadly feel his deficiency; and still more deplorable, his patients must suffer terribly on account of his ignorance. And how lamentably below a respectable professional position must he stand, who ignores this information, and makes manifest his ignorance to men of science, and the world at large.

However much we may pride ourselves on the high standing of our chosen calling, it can not be denied, that it is looked upon as occupying an inferior place in the Temple of Science, by those who practice exclusively the Healing Art.

The reason is quite obvious. *Our* labors are in a similar field to their own. Learning of a particular kind is necessary to work successfully in that field. Our co-laborers know that heretofore, at least, this knowledge has not been *generally* diffused among ourselves, and this fact was so patent to all, that a high degree of respect could not be awarded to those who neither had, or desired, that wisdom which would place them on a level with men of science. Individuals are not always accorded the respect which their merit deserves; adventitious circumstances may sometimes keep really great and worthy minds in the shade; but this can hardly be said of a profession whose rapid advancement is paraded for the admiration of the world. Whatever merit it possesses, above mere mechanism, will doubtless be readily appreciated by the scientific portion of the community.

Now Brethren, as honest men, what is our *duty?* How shall we remedy this great evil? Is not the way plain, if not comparatively easy? Let every one in the Dental Profession endeavor to perfect himself in *Anatomy*, *Physiology* and *Pathology*, on which all sound knowledge of *Therapeutics* must be based. Without these pre-requisites, the Healing Art must be one of mere routine and empirical practice. Have we not all received gifts from Science? Let us lay ourselves under still greater obligations, for the more we receive, the more able we are to repay the debt; and it should be our ambition to repay it with usury.

Our Dental Colleges present all the necessary facilities for acquiring the knowledge which our Profession *demands*, and we should, by all means, *insist* on our Students availing themselves of these high privileges; and if refused, should reject such men as unworthy members of an honorable profession.

Dental Therapeutics embrace the treatment of very few diseases, but the intimate relations which these sustain with those which afflict other parts of the system, call for extensive reading and study.

Chorea, or St. Vitus dance, is often a result or concomitant of tooth-ache, and is sometimes an immediate result of extraction. So also is temporary paralysis a result of toothache. Cramps in the stomach are caused by Periostitis. Scurvy, a constitutional disease, is first perceived in the gums; and the Dentist may be the first medical man called upon to treat it. What an awkward position would he hold, should his remedies applied or prescribed be local or surgical instead of general, specific, or constitutional.

Both Maxillary and Dental Periostitis are a frequent result of Syphilitic poison. How humiliating would be the discovery, especially if made by an enemy, that we had failed, from ignorance of its peculiar character, to treat such a case with ordinary intelligence. Is it any consolation to know that dignified M. D's. sometimes make similar mistakes?

This morning I received a call. Patient, a boy four years old. Has never taken mercury. For the last two months has eaten freely of fresh pork, sausages and mince pies. Symytoms for a week past and *present*: Gums dark red and swolen, bleed at the slightest touch; fetid, viscid, sanious discharge from alveolar border; wets his pillow with large quantities of grumous saliva; subdued fever. Has had a Physician who prescribed—what do you think! Borax, as a topical lotion! And this was all. I should be ashamed of the Iowa State Dental Society if I supposed that one of its members could exhibit such a marvelous degree of ignorance. Mercurious Vivus was prescribed for that child, and will cure him in less than a week. But, Brethren, the ignorance or carelessness of others is no excuse for our own. Let us severely examine *ourselves* and be sure that we are not found wanting.

There has always been so many Interlopers in our profession; men without education or scientific attainments; men whose sordid minds never look beyond a *Fee*, that the *Public* has come to regard us as mere Mechanics; and many are disposed to consider it a good joke when an internal remedy is prescribed for a dental disease. Last week a lady, aged 20, called upon me and described with much anxiety and excitement a severe tooth-ache which had lasted for eighteen hours. She was using bitartrate potassa to clean her teeth, when pain suddenly commenced in a bicusped plugged with "Wood's Alloy," and had continued without intermission since. She was much surprised, and certainly pleased when I assured her that I expected to save her tooth; but she smiled with incredulity when I prescribed Mercurius 3d dec. 1 gr'n every two hours; ten powders; and requested her to report next day. Patient reported third day *cured*, but said her friends laughed at her for taking medicine for tooth-ache· Gentlemen, let us make such cures so common, that they will be *expected*. When available, medical means should always be resorted to, in preference to surgical, for restoring health to any organ. In *this* case was it not much better than to have removed the plug and destroyed the pulp?

All works on Dental Therapeutics, so far as I know, are based on the Allopathic mode of practice. While I would accord to this School due honor for its antiquity and the good it has, and is performing in the world, yet I believe there is a *better way*. My personal experience for the last fifteen years has confirmed me in the belief of Hannemann's great principle:

Simillia, Simillibus Curantur.

This is a *truth* to which I believe all the medical world is destined to bow, as it is the great central luminary, around which, all other medical truths must revolve. The quantity of medicine necessary to produce therapeutic results is a mere matter of experience, and has nothing to do with the Great Principle itself. That infinitessimal doses have curative effects can not, at this late day be successfully denied. To my known opinions as a Homeopath, I attribute the assignment of the present theme to myself for an Essay. I shall, therefore, make no apologies for treating the subject from a base different from those who have preceded me.

A comprehensive dessertation on Dental Therapeutics would require a volume for its elucidation, and much more time than I can at present command, even if I was capable of doing justice to the whole subject; moreover, I do not think you require or expected it.

I shall attempt no more than to give you my *experience* with a very few remedies in their curative influence over a limited number of diseases peculiar to the dental organs. I will not take up your time with a description of these maladies, with which you are all familiar, but proceed at once to a brief pathogenesis of the medicines, and the symptoms which call for their therapeutical application.

Pathogenesis and curative symptoms of Mercurius Vivus, Carbo Vegetabilis, Iodinum, Iodide Potassium, Hepar Sulphuris, Aconitum, Arsenicum, Creosotum, Sulphur.

I do not pretend to give you here, anything like a full pathogenisis of the following remedies, having only noted those symptoms relating more particularly to the teeth and their surroundings. For a full description of the drugs, I must refer you to the books.

MERCURIUS VIVUS.—Dose 1 gr., 3d dec. trit., Increased activity and inflammation of salivary glands. Severe shooting pains in teeth and jaws. Teeth sore on percussion, and loose. Face-ache. Soreness over region of Antrum. Swelling of the face and gums. Inflammation of the gums; retraction of the same. Scurvy of the gums; they bleed easily; Sanious, purulent and viscid discharge from them. Putrid smell of the breath. Apthous, ulcerated, vescular and inflammatory condition of mucous membrane in any part of the mouth, tongue, cheeks, gums, or tonsils. Necrosis of maxillary bones and teeth. Pains aggravated by exposure to cold air, and at night; low fever; exhausting sweats; nausea and excessive prostration; bruised and sore feeling in all the bones; rheumatic and syphilitic symptoms.

MERCURY stands *pre-eminent* among dental remedies. It is the *Great Polychrest.* It seldom disappoints us, and its indications are rarely obscure. I have no time to write, neither have you to listen to a panegyric on the wonderful virtues of this ancient, powerful, and much abused and much maligned remedy. May your experience with it prove to be as happy as my own.

Carbo Vegetabilis.—Dose 1 gr., 3d dec. trit. Inflammation of the gums; ulceration and shrinking of same; scurvy of gums; sanious and viscid discharge from festoons; mercurial symptoms; all aggravated by open air and exercise. Great susceptibility to thermal changes. Typhoid symptoms Cold and blue appearance of face; cold sweats.

Iodine.—Dose, 1 drop, 3d dec. dil. Inflammation, atrophy and softening of the gums; Increased activity of salivary glands; inflammation, induration and ulceration of the same. Scrofulous symptoms.

Iodide Potassium.—Dose, 1 gr., 3d dec. trit. Syphilitic symptoms. Rheumatic diathesis; inflammation and ulcerative absorption of alveolar and maxillary bones; necrosis; swolen and purple gums; stimulation of salivary glands; periostitis.

Hepar Sulphuris.—Dose, 1 gr., 3d dec. trit. Mercurial and scrofulous conditions; inflammation and ulceration of salivary glands; purulent discharges from antrum, alveoli, and gums; teeth painful on occlusion of jaws; erysepitatous swelling of face. Particularly useful to remedy evil effects of mercury, or when the latter fails to cure.

Arsenic.—Dose, 1 gr., 3d dec. trit. Offensive, sanious discharges from the dental pulp, tongue, cheeks and gums, the latter purple and cold to touch; small ulcers on mucous membranes of buccal cavity; salivary glands excited and inflamed; Metallic taste in the mouth; suffering aggravated at night and at rest; teeth very sensitive to cold air and sore to touch; alveolo-dental Periostitis.

Aconite.—Dose, 1 drop, 3d dec. dil. Burning fever; heat preceded by chill; great thirst; congestion of dental pulp; throbbing pains in the teeth, which are relieved by topical bleeding.

Creosote.—Dose, 1 drop, 3d dec. dil. Excoriations of mucous membranes; hypertrophy of dental pulp; fungous growth of festoons of gums; fetid purulent discharges from old roots of teeth; apthous ulcerations of buccal cavity.

Sulphur.—Dose, 1 gr., 3d dec. trit. Great sensitiveness of dental pulp to thermal changes. Symptoms of diseases aggravated at night, also in the open air, and in draughts of air; excoriation of mucous membranes, apthae, inflammation of gums, induration and purulent secretions from the same.

Surgical means and topical applications are often called for in the treatment of the following diseases, but I shall only speak of *internal* remedies. In the choice of a medicine, care must be taken to get a *picture* of the disease, by noting all the symptoms present, and comparing this with the pathogenesis of different medicines. The pathogenesis of a drug is the picture of its symptoms. That drug which most resembles the disease, is a specific remedy for the latter and will not be likely to disappoint you.

In the treatment of Alveolo Dental Pereostitis, Mercury is indicated in the following conditions: severe throbbing pain; chronic or sub-acute inflammation; teeth sore on percussion; feel loose, in connection with general cold; with increased activity or inflammation of salivary glands; swelling of the gums; prosopalgia; high sthenic condition of the system; healthy pus exudations.

Give Iodide of Potassium for syphilitic causes; mercurial and strumoras diathesis, in connection with alveolar necrosis; purulent discharges; persistent continuance of disease unchecked by mercury; arsenical poisoning.

We give Sulrhur whenever the well chosen remedy fails to cure in this or any other disease, one or two doses, and then renew the *specific* medicine.

Hepar Sulphuris for scrofulous symptoms; abuse of mercury; viscid, muco-purulent exudation from margin of gums. The poison of syphilis is so extensively diffused throughout the world, that we must always be on our guard against its pernicious influence in modifying and rendering obstinate the disease under treatment.

*Alveolar Abscess.*—MERCURY is our first and best remedy, unless it is the cause of the disease. With swollen face and glands.

IODIDE POTASSA. in suspected syphilitic diathesis.

IODINE.—Unmistakeable scrofulous diathesis. As an injection, 10 drops to 100 of Alcohol.

CREOSOTE.—Injection as above.

HEPAR SULPHURIS, if caused by abuse of mercury.

*Inflammation of Antrum.*—MERCURY in either acute or chronic cases, when not caused by this drug. Prosopalgia; purulent secretions; swollen face; soreness of bicuspeds and molars on affected side. Especially useful when one has taken cold. With increased activity of salivary glands.

IODIDE POTASSA.—In syphilitic complications, or when mercury has failed. Rheumatic diathesis.

HEPAR SULPHURIS, when *caused* by mercury; in *abundant* purulent discharges; in scrofulous subjects; with general catarrhal symptoms.

IODINE AND CREOSOTE.—As injection, either combined or singly, 10 drops to ounce of alchohol, in profuse purulent discharges.

SULPHUR.—At the end of cure, or at any time during treatment of these diseases, when the specific remedy fails to ameliorate symptoms.

*Stomacace.*—MERCURIUS.—Seldom any other remedy is required. Inflammation and apthous ulceration of whole buccal cavity; gums red and swollen; festoons bleed easily; some appetite; feverishness; offensive, sanious and viscid discharges from festoons of gums; salivary glands excited or inflamed; discharging ropy offensive saliva; *false* mercurial symptoms; looseness of bowels, with griping pains.

ARSENIC.—Great pain in tongue, which is swollen and covered on its anterior margin, with small white ulcers; no appetite; great anxiety and prostration of vital powers; much thirst; burning pains in stomach and bowels; diarrhea.

CARBO VEGETABILIS.—Asthenic condition of system, caused by low diet, or damp, unhealthy habitations; abuse of mercury; effects of quinine disease, or intermittant fever.

SULPHUR.—Sour smell of mouth; alternate constipation and diarrhea.

*Inflammation of Gums.*—MERCURIUS.—Acute or chronic cases; purulent, viscid, sanious secretions; ulcerative absorption. Rheumatic causes.

CARBO VEGETABILIS.—Scurvy; profuse bleeding: depressed vital powers; typhoid symptoms.

IODIDE POTASSA.—With ulcerative absorption, and looseness of teeth; in rheumatic and syphilitic constitutions.

HEPAR SULPHURIS, for abuse of mercury; and scrofulous symptoms.

ARSENIC.—Black, cracked and cold condition of gums; offensive and sanious discharges; gums bleed easily; small white ulcers on mucous membrane, with excessive debility.

We give ACONITE in this and all other diseases when there is high fever.

*Inflammation of Salivary Glands.*—MERCURY is the usual specific.

IODINE is indicated when the cervical glands participate.

In acute cases of disease we give remedies once in one, two or three hours. In chronic cases, once, twice or thrice daily, one hour before eating. Particular attention should be paid to diet and regimen.

FORBIDDEN.—Coffee, teas, spirits, pickles, all kinds of condiments, spices, salted and smoked meats, pastries, confections, old cheese, pork, smoked or salted fish, perfumery and tobacco.

ALLOWED.—Wheaten bread, and plain farinaceous articles, fresh beef, mutton, uncooked shell-fish, wild game, potatoes, ripe fruits, fresh butter, milk, weak black tea, water, barley, rice and toast waters and gruels.

I have preferred to render myself liable to the charge of *repetition*, rather than leave the subject *obscure*, which I hope I have not done. And if what I have said shall cause *one*

of you, my brethren, to faithfully adopt this *system* of treatment, I shall feel amply repaid for my trouble.

